# LDCT screening for lung cancer in East Asian never-smokers: balancing benefits and overdiagnosis-related harms

**DOI:** 10.1016/j.lanwpc.2026.101877

**Published:** 2026-05-19

**Authors:** Xuan Xu, Haiquan Chen, Wanghong Xu

**Affiliations:** a Department of Epidemiology, Fudan University School of Public Health, 138 Yi Xue Yuan Road, Shanghai 200032, China; b Department of Thoracic Surgery, Fudan University Shanghai Cancer Center, 270 Dong An Road, Shanghai 200032, China; c Yiwu Research Institute, Fudan University, Building V of Zhongfu Square, Yiwu 322000, China

**Keywords:** Lung cancer, Never-smokers, East Asia, Low-dose computed tomography, Opportunistic screening, Overdiagnosis, Nodule management

## Abstract

East Asian never-smokers exhibit higher lung cancer incidence than their Western counterparts and account for roughly 40% of regional cases; nevertheless, they remain largely excluded from organised LDCT screening guidelines due to the absence of randomised evidence. Meanwhile, opportunistic LDCT has proliferated across East Asia, improving early detection while raising concerns regarding overdiagnosis. In this Viewpoint, we argue that ecological estimates may overstate overdiagnosis by overlooking evolving non-tobacco exposures and therapeutic advances, whereas targeted LDCT for well-defined high-risk never-smokers may yield net benefit if indolent lesions are managed conservatively. We therefore advocate for a risk-stratified, harm-conscious approach that avoids both indiscriminate expansion and categorical refusal, and propose four priorities: (i) population-specific risk stratification to refine screening eligibility; (ii) terminological reclassification of indolent lesions to reduce psychological and clinical burden; (iii) optimising the “surgical curative window” for early lung adenocarcinoma to minimise overtreatment; and (iv) accelerated randomised trials to inform East Asian-specific guidelines.

**Search strategy and selection criteria**

We searched PubMed, Web of Science, Scopus, and major Chinese databases (CNKI, Wanfang Data, SinoMed/CBM) for articles published between 1 Jan 2000 and 10 Feb 2026, supplemented by targeted Google Scholar searches. Our search strategy combined controlled vocabulary and free-text keywords across five thematic domains: (1) lung cancer in East Asian never-smokers (“lung cancer”, “pulmonary neoplasm”, “lung adenocarcinoma”, “never smoker”, “nonsmoker”, “East Asia”, “China”, “Japan”, “Korea”, “Taiwan”, “Hong Kong”); (2) low-dose CT (LDCT) and opportunistic screening (“low-dose computed tomography”, “LDCT”, “CT screening”); (3) overdiagnosis and epidemiological trends (“overdiagnosis”, “incidence”, “mortality”, “stage shift”); (4) risk stratification and decision modelling (“risk model”, “risk score”, “cost-effectiveness”, “ICER”, “Markov”); and (5) management of screen-detected lesions (“pulmonary nodule”, “ground-glass opacity”, “subsolid nodule”, “Lung-RADS”, “surveillance”, “segmentectomy”, “curative window”). We reviewed all eligible records and manually screened reference lists of key papers to identify additional studies. We primarily included English- and Chinese-language peer-reviewed publications, prioritising prospective cohorts, randomised trials if available, registry-based analyses, systematic reviews and meta-analyses, guideline and consensus documents, and decision-analytic evaluations; very small case series were generally excluded unless highly informative. Japanese- and Korean-language sources were also considered when they provided relevant primary evidence, identified through reference-list screening and targeted browsing of national agency and professional society websites. As a Viewpoint, we did not conduct formal risk-of-bias assessment; instead, we prioritised studies with well-defined inclusion criteria, explicit outcome measures, and adequate follow-up duration, cross-referencing key findings across multiple sources where possible. We additionally sourced trend data and policy background from reputable non-peer-reviewed repositories and governmental databases, including OECD health statistics, IHME Global Burden of Disease, IARC/WHO resources, the CONCORD programme, NCI SEER, national cancer registry outputs and health-agency reports from East Asian countries, and guidelines from professional bodies such as the Fleischner Society, the ACR Lung-RADS, and the IASLC.

## Introduction

Globally, lung cancer among never-smokers now accounts for 15–20% of all diagnoses,^1^ whereas in East Asia this proportion approaches 40%,^2^ with adenocarcinoma comprising 60–80% of these cases.^1^ This epidemiological shift parallels declining male smoking prevalence and the corresponding rise in disease among never-smoking women.

Western randomised trials (NLST and NELSON) have established that low-dose computed tomography (LDCT) reduces lung cancer mortality; however, benefit has been demonstrated exclusively in ever-smokers with at least 20 pack-years. In the absence of formal guidance for never-smokers, opportunistic LDCT programmes irrespective of smoking status have progressively expanded across East Asia. This sustained demand reflects modest scan fees (US$30–80), same-day availability, volume-based incentives embedded within routine health-check packages, and limited risk-assessment literacy among both clinicians and the public.^3^^,^^4^ These initiatives detect early-stage adenocarcinoma in never-smoking women, with reported post-resection survival exceeding 90%.^4^^,^^5^ Nevertheless, over 70% of screen-detected lesions present as indolent ground-glass opacities (GGOs), suggesting that many may never progress to life-threatening disease.

Although these observational findings cannot replace randomised evidence, they support the biological plausibility and potential utility of selective LDCT screening in never-smokers. The key question is therefore no longer whether to screen this population, but how to align expanding LDCT practice with emerging evidence to maximise benefit while minimising overdiagnosis and related harms. Here, we argue for moving beyond the simple yes-or-no debate towards a pragmatic approach: targeted LDCT for well-defined high-risk never-smokers, based on refined risk stratification, conservative lesion management, and stronger evidence.

## Widening gaps in evidence, guidelines, and practice

International guidelines currently restrict LDCT screening to individuals with ≥20–30 pack-years of smoking history. This cautious approach is grounded in Western population data: in a population-based study from Western Switzerland, the 10-year risk of lung cancer among never-smokers remained below 0.5%^6^; in the Prostate, Lung, Colorectal and Ovarian Cancer Screening Trial (PLCO), the highest observed 6-year risk among 65,711 never-smokers was 1.47%, still below the commonly used 1.51% screening threshold^7^; and the Microsimulation Screening Analysis (MISCAN) modelling suggests that 6162 average-risk never-smokers would require screening to avert one lung-cancer death, compared with 353 screens required for US Preventive Services Task Force (USPSTF)-eligible ever-smokers.^8^ No randomised trial has demonstrated a mortality reduction in this population, and the frequent detection of GGOs raises understandable concern regarding overdiagnosis.

East Asia, however, presents a markedly different risk landscape, where lung cancer in never-smokers is particularly prominent and age-standardised incidence among non-smoking women is more than twice that in Western populations.^9^ Consequently, applying identical pack-year thresholds may substantially underestimate both absolute benefit and cost-effectiveness. Observational evidence is accumulating quickly in this region. A Japanese national cohort of 12,114 participants demonstrated that restricting eligibility to ≥30 pack-years would miss 72.9% of screen-detected cancers.^10^ Similarly, the prospective Guangzhou LUNG-CARE project, which offered LDCT irrespective of smoking history, reported a 1.7% detection rate, with 82.5% of malignancies identified at stage I, substantially reducing missed diagnoses compared with conventional high-risk criteria.^11^

**Fig. 1:**
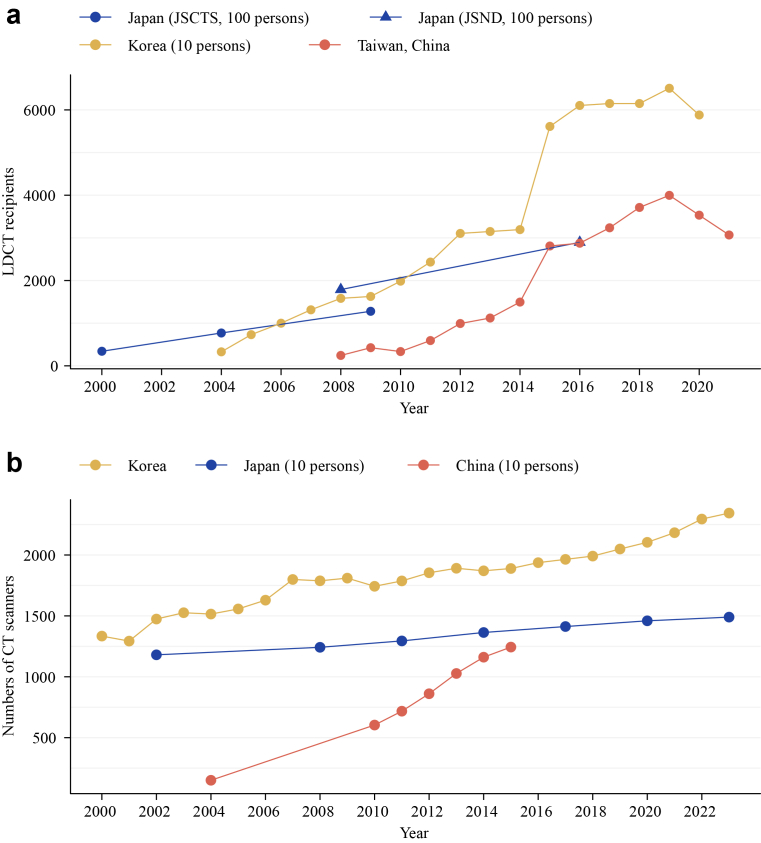
**Surging uptake of LDCT screening in East Asian populations.** (a) Annual volumes of LDCT screening in Japan (national estimates: JSCTS 2000/2004/2009; JSND 2008/2016), South Korea (national self-paid health check-up data 2004–2020 from NHID and 11 academic centers),^3^ and Taiwan, China (single-center data 2008–2021 from Kaohsiung Veterans General Hospital).^17^ Data were rescaled for cross-source visual comparability. (b) Annual CT scanner counts: Korea and Japan (OECD Data Explorer 2000/2002–2023) and mainland, China (NHC 2004–2015). Abbreviations: LDCT, low-dose computed tomography; JSCTS, Japanese Society of CT Screening; JSND, Japan Society of Ningen Dock; NHID, Korea National Health Information Database; OECD, Organisation for Economic Co-operation and Development; NHC, National Health Commission of the People's Republic of China.

In practice, opportunistic screening has become the de facto standard. In 2023 alone, LDCT was incorporated into more than half of health check-up examinations within a large national dataset in China,^12^ against a backdrop of approximately 524 million preventive health-check visits nationwide.^13^ In tertiary hospitals in Shanghai and Beijing, over half of never-smoking check-up attendees now request the examination.^14^^,^^15^ At Weihai Municipal Hospital Healthcare Group, 42.9% of the 5234 lung cancer patients diagnosed between 2016 and 2021 were identified through opportunistic LDCT that did not consider smoking history.^16^ Rising outpatient demand and expanding scanner capacity suggest continued regional uptake (Fig. 1).^3^^,^^17^

In the absence of randomised evidence, major guidelines currently do not recommend routine LDCT screening for never-smokers, generally restricting it to research settings. Nonetheless, several Chinese expert-consensus statements have progressively broadened eligibility criteria by incorporating secondhand smoke, occupational exposure, or family history of lung cancer as core risk factors,^18^ effectively endorsing screening for selected high-risk never-smokers.

## Overestimation of overdiagnosis in ecological studies

Cancer overdiagnosis is generally defined as the detection of cancers that would not become clinically evident or cause death during a person’s lifetime if left undiscovered. Clinically, this leads to unnecessary treatments; in the LDCT screening context, it implies that such individuals would not have been labelled as having lung cancer or recorded in cancer registries without screening. With no randomised trial available to quantify overdiagnosis in East Asia, current assessments rely largely on qualitative evaluation and ecological inference: rising incidence coupled with stable mortality, and increasing early-stage disease without corresponding late-stage decline, are commonly interpreted as suggestive of overdiagnosis (Table 1).^3^^,^^17^^,^^19^, ^20^, ^21^, ^22^, ^23^ However, incidence reflects both cumulative risk exposure and screening intensity, whereas mortality depends on incidence, stage distribution, and treatment advances (Table 2), confounding simple ecological interpretations.

**Table 1:** Evidence for overdiagnosis in LDCT screening for lung cancer in East Asia.

First author, published year	Study design	Study period	Population	Participants	Smoking prevalence (%)	Evidence for overdiagnosis
Sone et al., 2007^19^	Single-arm	1996–1998	General population in Nagano, Japan	5480 residents aged 40–74 years	2047 (37.4%)	Post-hoc analysis estimated overdiagnosis at 13.3% overall (6 of 45 cases) and 40% for non-solid lesions.
Goo et al., 2022^20^	Ecological	1999–2019	General population in Korea	Nationwide lung cancer statistics from the Korea National Cancer Incidence Database	1998–2018: men 66.3%→36.7%; women 6.6%→7.5%	Among women, incidence increased by 35% (12.5→16.9 per 100,000) while mortality decreased by 26% (9.5→7.0); early-stage incidence increased by 128% (2.7→9.1) whereas late-stage increased by 24% (7.8→9.7). Suggestive of overdiagnosis.
Kim et al., 2025^3^	Ecological	1999–2022	General population in Korea	Entire Korean population	2001–2021: men 61.3%→30.7%; women around 6%	Among women, incidence increased (29.8→39.3 per 100,000) while mortality decreased (23.3→19.8); localised-stage incidence nearly doubled (7.6→13.7) whereas distant-stage decreased only slightly (16.1→15.0). Suggestive of overdiagnosis.
Gao et al., 2022^21^	Ecological	2004–2018	Female adults in Taiwan, China	Approximately 12 million Taiwanese women	1980–2018: women <5%	Among women, early-stage incidence increased more than sixfold (2.3→14.4 per 100,000) without a corresponding late-stage decline (18.7→19.3). Suggestive of overdiagnosis.
Chen et al., 2024^17^	Ecological	2008–2021	Hospital-based adults in Kaohsiung, Taiwan, China, with self-paid LDCT mainly offered to those aged 40–80 years	4971 lung cancers from cancer registry at Kaohsiung Veterans General Hospital; linked with LDCT imaging database	2009–2021: 50.0%→34.3%	Increasing LDCT volume paralleled rising stage 0 diagnoses while stage IV disease remained stable; screening was associated with stage 0 (OR 7.6) and stage 0–I disease (OR 17.1). Suggestive of overdiagnosis.
Wang et al., 2023^22^	Ecological	2002–2017	General population in Pudong New Area of Shanghai, China	Approximately 3 million residents in Pudong New Area of Shanghai	women <5%	Among women, incidence increased (APC 11.98%) while mortality decreased (APC −1.27%); early-stage incidence increased 19-fold (0.9→17.0 per 100,000) without a corresponding late-stage decline (9.1→8.5). Suggestive of overdiagnosis.
Xie et al., 2025^23^	Ecological	2002–2020	General population in Pudong New Area of Shanghai, China	Approximately 3.21 million residents in Pudong New Area of Shanghai	2007–2020: overall 30%→20%; men 62%→38%; women <3%	Among women, incidence increased (19.20→47.69 per 100,000) while mortality decreased (15.93→13.16); early-stage incidence increased 15-fold (1.22→19.09) without a corresponding late-stage decline (8.50→9.01); model-estimated overdiagnosis increased from 22% to 50%, with an overall estimate of 40%.

LDCT, low-dose computed tomography; OR, odds ratio; APC, annual percentage change.

**Table 2:** Influence of key determinants on lung-cancer incidence and mortality.

Lung cancer	Changes induced by				Observed trends
	Risk exposure	Early detection	Overdiagnosis	Advanced therapy	
Overall incidence	↑	–	↑	–	↑
Early-stage incidence	↑	↑	↑	–	↑
Late-stage incidence	↑	↓	–	–	Uncertain
Survival rate	–	↑	↑	↑	↑
Mortality	↑	↓	–	↓	Uncertain

**Fig. 2:**
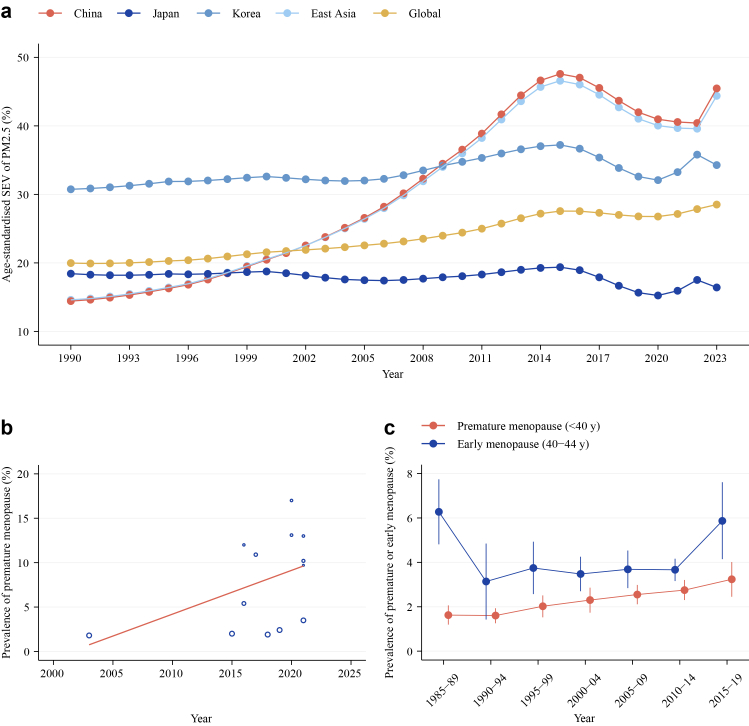
**Secular trends in ambient particulate matter pollution and premature reproductive senescence.** (a) Ambient particulate matter exposure (SEV) (both sexes combined), age-standardised, GBD, 1990–2023. (b) Premature menopause prevalence, global, 2003–2021.^35^ Solid line denotes pooled meta-regression fit across 13 studies. (c) Premature and early menopause prevalence in LMICs, 1985–2019.^36^ Abbreviations: SEV, summary exposure value; GBD, Global Burden of Disease; LMICs, low- and middle-income countries.

While most non-tobacco risk factors have plateaued or declined over the past decade, ambient PM_2.5_ and many non-asbestos occupational carcinogens continue to increase.^24^ Each 10 μg/m^3^ increment in PM_2.5_ correlates with a 17–32% higher incidence of lung cancer among never-smokers, particularly adenocarcinoma in women.^25^^,^^26^ In Taiwan, age-standardised adenocarcinoma rates nearly tripled between 1995 and 2015, tracking the secular increase in PM_2.5_ concentrations.^27^ Lung cancer in never-smokers disproportionately affects women, with reproductive and hormonal factors increasingly recognised as contributors. Premature menopause, in particular, has been consistently associated with elevated lung cancer risk,^28^, ^29^, ^30^, ^31^ consistent with the hypothesis that reduced lifelong exposure to endogenous estrogens may diminish the putative protective effects of these hormones against lung carcinogenesis.^32^, ^33^, ^34^ Concurrent rises in lifetime PM_2.5_ exposure and premature menopause prevalence have been reported, with the steepest increases in low- and middle-income countries (Fig. 2).^35^^,^^36^ These patterns plausibly contribute to the sustained rise in lung cancer incidence.

**Fig. 3:**
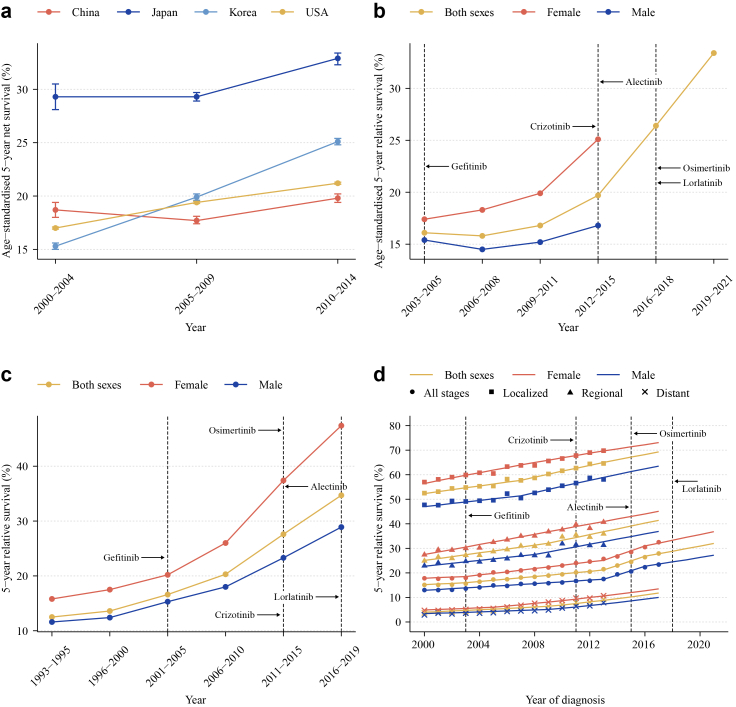
**Five-year survival trends in lung cancer: overall and stage-specific patterns following novel therapy adoption.** (a) 5-year net survival (age-standardised), China/Japan/Korea/US, CONCORD-3, 2000–2014. (b) 5-year relative survival (sex-specific, age-standardised), China, 2003–2021.^40^^,^^41^ (c) 5-year relative survival (sex-specific), Korea, 1993–2019.^42^ (d) 5-year relative survival (stage- and sex-specific), SEER, the US, 2000–2022. Abbreviations: CONCORD, global surveillance of cancer survival; SEER, Surveillance, Epidemiology, and End Results; US, the United States.

Prognosis remains tightly linked to stage at diagnosis. The 2025 IASLC staging manual cites 82% 5-year survival for Stage IA non-small cell lung cancer (NSCLC) vs. 7% for Stage IVB.^37^ Over the past decade, targeted therapies have transformed outcomes in molecularly selected advanced NSCLC, extending median survival among never-smokers from 8–12 months to 3–5 years and accelerating population mortality declines since the mid-2010s.^38^^,^^39^ Notably, survival improvements manifest 2–3 years after each therapeutic advance, predominantly in advanced disease (Fig. 3).^40^, ^41^, ^42^

Because guideline-endorsed LDCT screening is restricted to high-risk ever-smokers, both lung-cancer incidence and mortality have declined in this group. This dual decline reflects reduced tobacco exposure, detection of clinically significant early-stage cancers, minimal overdiagnosis within this stratum, and incremental therapeutic gains that reduce case fatality rates. Extending LDCT to never-smokers, a practice already prevalent in East Asia,^3^^,^^4^ would inevitably reveal a larger reservoir of indolent adenocarcinomas, thereby amplifying recorded incidence. Should non-tobacco exposures remain stable, this increment would represent pure overdiagnosis. However, if ambient PM_2.5_, radon, or other non-tobacco risk factors continue to rise, the excess would comprise both overdiagnosed indolent lesions and true exposure-attributable cancers. Stage migration and modern therapy would prolong survival in either scenario, potentially attenuating, but not preventing, a subsequent mortality rise attributable to increasing risk; should population risk continue to climb, mortality could ultimately plateau or even increase after a lag period.

Lung cancer trends in Pudong New Area of Shanghai illustrate these dynamics.^23^ During 2002–2010, a period essentially devoid of LDCT screening, male smoking prevalence dropped from 66.0% to 52.9%,^43^^,^^44^ and lung cancer incidence and mortality declined in tandem, confirming tobacco control as the dominant driver. Among women (smoking rate <3%), early-stage incidence remained stable until 2011, then surged following widespread opportunistic LDCT uptake, while late-stage incidence began to decline, a classic screening-induced stage shift. Female adenocarcinomas exhibited a more pronounced pattern: the steady pre-2011 rise reflected increasing non-tobacco risk exposures, whereas post-2011 acceleration represented superimposed detection of indolent tumours upon this baseline. Notably, the steep pre-2011 mortality decline lost statistical significance thereafter, highlighting the competing influences of rising incidence, stage migration, and incremental therapeutic gains.

Collectively, rising incidence likely stems from overdiagnosis alongside genuine increases in exposure-related cancers. Stable or declining mortality, meanwhile, reflects stage migration and therapeutic gains, though this trend may reverse if exposures continue to climb. Using incidence–mortality divergence alone to estimate population-level overdiagnosis therefore risks overestimation, as it ignores both shifting risk profiles and improving treatments.

## Emerging evidence supporting a high-risk strategy

Randomised evidence regarding LDCT screening in never-smokers remains limited, with ongoing trials in Japan and China yet to mature.^45^^,^^46^ Meanwhile, accumulating observational data demonstrate improved stage distribution and survival gains,^10^^,^^15^^,^^47^, ^48^, ^49^, ^50^, ^51^, ^52^ providing preliminary evidence of potential mortality benefit in never-smokers with well-defined risk profiles (Table 3).^53^, ^54^, ^55^ The prospective TALENT study in Taiwan enrolled 12,011 never- or light-smokers aged 55–75 years with at least one additional risk factor, most commonly family history of lung cancer. At 1-year follow-up after baseline LDCT, lung cancer was identified in 2.6% of participants (2.1% were invasive), with 77.4% diagnosed at stage I.^48^ Building upon these findings, Taiwan launched a biennial programme in 2022 offering LDCT to never-smokers with first-degree family history. Interim analyses showed a 1.2% detection rate, with 85% of cancers diagnosed at stage 0–I and a 77% reduction in stage II–IV disease compared with the pre-programme period, projecting a 55% decline in lung-cancer mortality.^56^ Although mature mortality follow-up remains unavailable, these findings lend stronger support that targeted screening in high-risk never-smokers can reduce advanced-stage disease and may ultimately translate into meaningful mortality benefit.

**Table 3:** Evidence for potential benefit in LDCT screening among never-smokers in East Asia.

First author, published year	Areas	Study period	Study design	Study population	Eligibility for screening	Participants	Never-smokers (%)	Benefit in never-smokers			
								Detection rate	Stage distribution	Survival	Mortality
Yu et al., 2024^53^	Liaoning, China	2012–2021 (median 5.25 years)	Prospective cohort	High-risk population	Age 40–74 years; high risk by sex-specific Harvard Risk Index (non-smoking men excluded)	58,136 (20,346 screened; 37,790 non-screened)	18,121 (31.2); 7182 (35.3) in screened group	–	–	–	Screened vs. non-screened: lung-cancer mortality HR 0.49 (0.20–1.19); all-cause mortality HR 0.69 (0.52–0.92)
Wang et al., 2024^47^	Zhejiang, China	2017–2020	Retrospective cohort	General population	Age 20–80 years; asymptomatic; no prior lung cancer	42,018	32,595 (77.6)	men 0.43%; women 0.85%	Stage IA: men 81.5%; women 93.2%	–	–
Tang et al., 2024^15^	Beijing, China	2006–2022 (median 25.4 months)	Retrospective cohort	General population	Age ≥18 years; asymptomatic; no prior lung cancer; fit for curative surgery; irrespective of smoking history	30,468	21,426 (70.3)	1.0%	Stage 0–I 78.8%; stage III-IV 4.2%	5-year survival 98.1%; 10-year survival 92.6%	–
Chang et al., 2024^48^	Taiwan, China	2015–2019 (1-year follow-up)	Prospective cohort	High-risk population	Age 55–75 years; never or <10 pack-years quit >15 years; ≥1 risk factor (family history, passive smoke, prior COPD/TB, cooking exposure)	12,011	11,201 (93.3)	Overall: 2.6%; invasive 2.1%	Overall: stage 0–I 96.5%; stage I 77.4%; stage IV 1.6%	–	–
Wang et al., 2023^50^	Taiwan, China	2007–2021 (median 12.0 years)	Prospective cohort	High-risk population	1st-/2nd-degree relative family history; age ≥55 years or older than proband age at onset if proband was ≤54 years	1102	771 (70.0)	5.2%; 9.4% in multiplex families vs. 3.7% in simplex families	Stage I 67.5%; stage IV 20.0%	Median overall survival not reached	–
Li et al., 2021^51^	Tianjin, China	2017–2020	Single-arm	General population	Age 40–74 years; no cancer history; no chest CT within 1 year	5523	3767 (68.2)	0.4%	Stage I 86.7%; stage IV 13.3%	–	–
Kim et al., 2020^54^	Seongnam, Korea	2009–2018	Retrospective cohort	General population	Age ≥18 years; asymptomatic; no prior lung cancer	37,436	17,968 (48.0)	0.47%	Stage I 89.3%	Better survival than ever-smokers	Lung cancer death: 2.4% in never-smokers vs. 12.2% in ever-smokers
Kang et al., 2019^52^	Seongnam, Korea	2003–2016	Retrospective cohort	General population	Adults undergoing LDCT regardless of smoking history; no prior lung cancer	28,807	12,176 (42.2)	0.45%	Stage I 80.0%	5-year overall survival 96%; 10-year overall survival 96%	Lung cancer death: 1/55 (1.8%)
Kakinuma et al., 2020^10^	Tokyo, Japan	2004–2017 (median 7.5 years)	Single-arm	General population	Age ≥40 years; no cancer diagnosis within 1 year and not receiving cancer treatment	12,114	6021 (49.7)	1.1%	Stage IA 90.9%	5-year cancer-specific survival 96.8%; 10-year cancer-specific survival 96.8%	–

LDCT, low-dose computed tomography; HR, hazard ratio; COPD, chronic obstructive pulmonary disorder; TB, tuberculosis.

These observations have prompted decision-analytic evaluations suggesting that a high-risk strategy may prove more efficient. Modelling in Hong Kong applying TALENT-derived risk profiles found annual LDCT for high-risk never-smokers cost-effective (incremental cost-effectiveness ratio [ICER] US$9610 per QALY), below local willingness-to-pay thresholds (US$24,302–40,202).^57^ Markov modelling in mainland China focusing on never-smokers with first-degree family history also supported targeted screening: biennial LDCT from age 55 yielded favourable ICERs (68,932 and 80,056 CNY per QALY for men and women) under the three-times-GDP threshold.^58^ Nationally, the NCC-LC_m2021_ model demonstrates superior performance of risk-based selection over simple eligibility criteria, reinforcing the pragmatic case for risk-targeted rather than indiscriminate LDCT screening.^59^

Thus, while overdiagnosis remains a legitimate concern, emerging data suggest potential net benefit in well-defined high-risk subgroups. Longer-term cohort studies with mature mortality endpoints and rigorous randomised trials are warranted to establish high-level evidence.

## Precise, dynamic, and equitable risk stratification

Across studies, the most consistently replicated risk factors among never-smokers include secondhand smoke, radon, ambient and household air pollution, occupational carcinogens, chronic pulmonary disease, and family history. While indoor exposures decline, ambient PM_2.5_ persists across East Asia (Fig. 2a), disproportionately affecting women^60^ and potentially interacting with genetic susceptibility to promote carcinogenesis.^61^ Notably, lung adenocarcinomas in East Asian never-smoking women show high frequencies of actionable driver mutations (EGFR, ALK, ROS1), indicating a distinct biological susceptibility.^1^ Family history and polygenic scores may enhance risk stratification. Emerging population data also identify “young, female, never-smokers” as high-risk for GGO-featured adenocarcinoma, challenging traditional screening paradigms.^62^ Women show greater lung-cancer susceptibility than men at comparable tobacco exposure, with never-smoking risk more than two-fold higher^63^^,^^64^; reproductive or hormonal factors, particularly premature menopause, are increasingly recognised as independent contributors to risk.^28^, ^29^, ^30^, ^31^ Future eligibility should shift towards precise, population-specific risk stratification integrating demographic, clinical, environmental, and genetic data.

However, identifying predictors is only the first step. Risk stratification must account for evolving risk profiles and regional heterogeneity. Traditional exposures such as household solid-fuel use and passive smoking, long considered major contributors in China,^65^^,^^66^ may decline in large cities due to cleaner fuels and smoking control, yet persist in non-urban settings with poor kitchen ventilation and occupational hazards. Women most exposed may also be least reachable through employer-organised or opportunistic screening. Stratification should therefore be periodically recalibrated to reflect changing exposure patterns, tailored to local epidemiology, and aligned with resource allocation and health equity. Emerging tools, including artificial intelligence, may help integrate multiple risk factors to support more individualised, implementable screening strategies for never-smokers.

## Management of screen-detected lesions

Overdiagnosis carries risks of overtreatment, pulmonary function impairment, psychological distress, and resource consumption. These concerns are amplified by the recognition that many screen-detected nodules, particularly pure GGOs, may never progress. Adenocarcinoma, the subtype most susceptible to overdiagnosis, exhibits the widest outcome spectrum: pure GGOs corresponding to in situ or minimally invasive disease confer nearly 100% five-year survival, whereas invasive tumours approximate 80% survival even at pathological stage I.^67^ These data indicate that not every stage I adenocarcinoma represents a futile diagnosis.

Modern management can mitigate some of these harms. Indolent lesions, particularly pure GGOs, are often managed through structured surveillance; when resection is indicated, minimally invasive, parenchyma-sparing techniques (VATS/robotic wedge or segmentectomy) are increasingly employed to preserve pulmonary function and shorten hospitalisation.^68^ Deferred resection for multifocal GGOs has proved safe,^69^ and observed survival advantages reflect genuine cure rather than lead-time bias, though complete long-term follow-up remains maturing.^70^ Rather than characterising every GGO as an imminent threat, it is more constructive to view these findings as manageable consequences of earlier detection.

For most subsolid nodules, surveillance is prioritised and escalated when radiological features suggest invasion. Screening protocols such as Lung-RADS recommend interval CT for persistent nonsolid or small part-solid nodules, with intensified management if the solid component enlarges and more invasive evaluation for suspicious lesions, ideally individualised through shared decision-making. This spectrum shifts the focus from overtreatment to vigilant surveillance, enabling most low-risk lesions to be managed safely in outpatient settings. However, surveillance also carries consequences: repeated imaging, radiation accumulation, patient anxiety, and residual uncertainty still impose downstream burdens.^71^ Tailored intervals, informed by dynamic risk reassessment and radiological stability rather than fixed annual CT, may therefore be more appropriate for never-smokers. These trade-offs underscore the need to define more precisely when surveillance remains appropriate and when resection offers durable cure benefit.

Accumulating evidence supports a “surgical curative window” for early lung adenocarcinoma. Resection within this window achieves durable cure, with ten-year recurrence-free survival approaching 100%.^72^^,^^73^ Management thus becomes a pragmatic balance between tumour biology and individual life expectancy: when residual life-years substantially exceed projected time to progression, resection offers the possibility of lifelong cure; when life expectancy is limited, active surveillance averts unnecessary surgery.

The immediate priority is refining imaging and biological markers to define this window with greater precision. High-resolution CT predicts pathological invasion with 83% accuracy; a solid component ≥6 mm represents a robust radiological threshold.^74^ Complementary molecular signatures, such as a two-gene (COL11A1 and THBS2) expression classifier, can retrospectively stratify stage I adenocarcinoma into indolent and progressive subtypes,^75^ though prospective validation remains pending. Conceptually, molecularly indolent tumours could be re-designated as pre-invasive lesions, analogous to colorectal adenomatous polyps, thereby reducing psychological and societal burden. Such reclassification remains investigational until international consensus incorporates molecular and histological criteria into formal nomenclature.

## Actionable priorities for East Asia

For East Asian never-smokers, the policy question has shifted from whether to screen to how to govern screening already in practice. We propose four priorities. First, replace indiscriminate LDCT with risk-stratified, population-specific eligibility. Second, manage indolent lesions, especially persistent pure GGOs, through conservative surveillance and terminological reclassification to reduce unnecessary burden. Third, refine the surgical curative window, reserving resection for high-benefit lesions while following stable, low-risk cases with tailored intervals. Finally, accelerate randomised trials to establish mortality benefit and inform region-specific guidance. Until then, the defensible path is high-risk, harm-conscious, continuously evaluated screening, not indiscriminate expansion.

## Contributors

XX, a doctoral candidate in cancer epidemiology, conducted systematic review of the relevant literature, performed the analyses, and drafted the initial manuscript. HQC, full professor of thoracic surgery, interpreted the operative data, ensured surgical accuracy and clarity, and critically revised the manuscript for intellectual content. WHX, full professor of epidemiology and guarantor, conceived the study, provided overall academic leadership, and critically revised the manuscript for intellectual content. XX and WHX directly accessed and verified the underlying source data used in the manuscript and figures. All authors had full access to all data reported in the manuscript, approved the final version, and accept responsibility for the decision to submit for publication.

## Declaration of interests

We declare no competing interests.

## Appendix A Supplementary data

Translated abstract
