# Supplementary material for: LDCT screening for lung cancer in East Asian never-smokers: balancing benefits and overdiagnosis-related harms
Source: Lancet Reg Health West Pac. 2026 May 19;70:101877. doi: 10.1016/j.lanwpc.2026.101877 (PMC13213730; doi:10.1016/j.lanwpc.2026.101877)
Supplement: Translated abstract [file mmc1.pdf]

东亚不吸烟者肺癌发病率显著高于西方人群，约占该地区肺癌病例的 40%。然而，由于缺乏随机对照试验证据，该人群目前被排除在有组织 LDCT 筛查指南之外。与此同时，机会性 LDCT 在东亚迅速普及，虽提升了非吸烟者肺癌的早期检出率，却也引发了对过度诊断的担忧。本文认为，生态学估算可能因忽视不断演变的非烟草暴露因素及治疗进展而高估过度诊断风险；若对明确界定的高危不吸烟者实施针对性 LDCT 筛查，并对惰性病灶采取保守管理，则可能实现净获益。因此，基于现有证据，我们倡导采取风险分层、注重获益-伤害比的策略，既避免盲目扩大筛查范围，也不一概拒绝非吸烟者参与，并提出四项优先事项：（i）建立人群特异性风险分层模型，优化筛查准入标准；（ii）对惰性病灶进行术语重分类，以减轻患者心理与临床负担；（iii）优化早期肺腺癌的“手术治愈窗口”，最小化过度治疗；（iv）加速开展随机对照试验，为制定东亚特异性指南提供循证依据。
